# Preparation, Chromatic Properties Analysis and Proportioning Optimization of Co-Cr-Fe-Based Black Pigment

**DOI:** 10.3390/ma16175785

**Published:** 2023-08-24

**Authors:** Weiran Zhang, Ziyu Li, Guohua Wu, Wei Wu, Hailan Zeng, Haiyun Jiang, Weili Zhang, Ruomei Wu, Qiong Xue

**Affiliations:** 1 School of Packaging and Materials Engineering, Hunan University of Technology, Zhuzhou 412007, China; jetwalkerz@gmail.com (W.Z.); ziyv.lee@gmail.com (Z.L.); wghaiyyr1120@163.com (G.W.); waqarrd@163.com (W.W.); zenghl0424@163.com (H.Z.); zh_weili@163.com (W.Z.); cailiaodian2004@126.com (R.W.); xueqiong2005@163.com (Q.X.); 2 National and Local Joint Engineering Research Center for Advanced Packaging Material and Technology, Hunan University of Technology, Zhuzhou 412007, China

**Keywords:** black ceramic pigment, response surface methodology, co-precipitation method, chromatic properties, spinel

## Abstract

The utilization of Co-Cr-Fe-based black pigments bears considerable significance within the realm of commercial ceramic pigments, owing to their distinctive spinel structure, remarkable high-temperature stability, and exceptional chromatic attributes. This study delves into the synthesis of diverse black pigment configurations by employing the co-precipitation method, leveraging the interplay of these three metallic oxides. This investigation encompasses a comprehensive scrutiny of ion valences, crystal structures and parameters, colorimetric properties, and their interrelationships. The methodology integrates the response surface methodology (RSM) framework, using theoretical formulations to navigate the material ratios and elucidating the associations between the resultant compositions and color coordinate values, aligned with the CIE-Lab* colorimetric methodology. The derived predictive models yielded an optimized black pigment composition, characterized by heightened black intensity and a refined formulation.

## 1. Introduction

Inorganic black pigments are an essential kind of pigment in the ceramic industry. Most of them contain transition metal elements, including cobalt, chromium, iron, nickel, manganese, copper, etc. [1,2,3]. Besides their intense tonality performance, inorganic black pigments are stable at high temperatures, even at 1360 °C or above, making them suitable for the fabrication of underglaze ceramics [4]. Due to the high temperatures during production, common colorants cannot maintain the satisfactory and reliable chromatic property. Recently, the most popular commercial black pigments used on porcelains, tiles, and ceramic bodies have been based on spinel structures [5,6,7] due to their high stability at high temperatures and under glazes, which aligns with the Dry Color Manufacturer’s Assn (DCMA) classification [8]. The typical commercial black pigments used in the ceramic industry consist of two basic spinels: iron-cobalt-chromite (Fe,Co)(Fe,Cr)_2_O_4_ (DCMA 13-40-9) and chrome-iron-nickel (Ni,Fe)(Fe,Cr)_2_O_4_ (DCMA 13-50-9) black spinels [9,10]. Due to environmental friendliness and health regulations, Ni is required to be removed from the recipes [11]. Mestre and co-workers [12] reported the synthesis and characterization of the Cr_2_O_3_–CoO–FeO black pigment, which was synthesized using the solution combustion method. Although much of the recent research has focused on the preparation, structures, additions, geometry, etc. [13,14,15,16], there is still a lack of sufficient knowledge regarding the specific relationship between the proportions of raw materials and chromatic properties. Thus, it is difficult and cumbersome to control the color in both laboratories and industries. The Cr-Co-Fe-based pigment was selected for research because this spinel-type pigment is a standard industrial black pigment and covers a wide chromophore range.

However, the design criteria of colorants are empirical and are related to cumulative light absorption by multiple chromophores [3]. Colors emerge through a process of subtractive synthesis, intricately influenced by the presence of chromophoric cation clusters [17]. Each of these clusters possesses a distinctive optical band, selectively absorbing a designated wavelength range within the visible spectrum. For many black colorants, such as Cr-Co-Fe pigments, they correspond to multiple metal elements, and changes in the content of each one will cause valence transformations and/or alterations in their crystalline structure, resulting in chromatic deviations. Thus, achieving ideal colorants, such as pigments with high black intensity, becomes challenging due to the uncontrollable nature of chromatic properties. Also, it is difficult to unify the color between different batches during the fabrication process of conventional ceramic black pigments. Chromatic aberration and unpredictable color have severely prevented the development of ceramic pigments and have led to increased industrial waste and energy consumption.

Response surface methodology (RSM) can be introduced to establish the statistical models and illustrate the mathematical relationships between the ratios of transition metals and color rending. RSM is a statistical method used for solving multivariate problems by analyzing the regression equation to find the optimal process parameters [18]. This encompasses systematic experimental design methodologies, precise data acquired from empirical investigations, and the utilization of several quadratic regression equations to aptly model the intricate functional correlation between independent factors and corresponding response values. This experimental condition-seeking method is suitable for solving nonlinear data processing problems. By applying regression to the process and plotting the response surfaces and contours, the response values corresponding to the levels of each factor can be easily found. Based on the response values at each factor level, the predicted optimal response values and the corresponding experimental conditions can be determined [19].

Here, we report a family of Cr-Co-Fe-based pigments prepared using the co-precipitation method. The co-precipitation method is highly significant in pigment preparation due to its capacity to enable controlled composition, morphology, and properties, ensuring consistent and tailored color outcomes for various applications. Then, the phases, valences, structures, and chromatic properties of the samples are characterized. The RSM technique is employed in research and plays a significant role in establishing specific contacts between the ratios of Co, Cr, and Fe and color coordinate values. The relationship models illustrate the impacts of the formulation on coloration and can help to adjust the ratio for achieving ideal color in this system. In addition, this paper takes other metal elements into consideration, such as aluminum, silicon, potassium, calcium, and sodium, because they are essential components of glaze, pottery billet, and commercial pigments, especially aluminum and silicon.

## 2. Experimental Procedure

### 2.1. Pigment Preparation

Black Co-Cr-Fe system pigments were synthesized using co-precipitation in order to guarantee fine and homogeneous slurry precipitation. Cobalt acetate (C_4_H_6_CoO_4_ · 4H_2_O, Aladdin Corp., Shanghai, China), chromite acetate (C_6_H_9_CrO_6_, Aladdin Corp., Shanghai, China), and ferric chloride (FeCl_3_· 6H_2_O, Aladdin Corp., Shanghai, China) were used as starting materials for pigment synthesis. In different batches, they were dissolved separately in water according to the designed ratio (Table 1), and then they were blended for the co-precipitation process. The formulations of the batches were developed based on spinel crystal theory and our knowledge [20,21]. Some excess contents were deliberately used for RSM. Ammonia water (NH_3_·H_2_O, Aladdin Corp., Shanghai, China) was added drop by drop under magnetic stirring at 500 rmp to control the pH value at around 9 or marginally above. The stirring process was continued for a while to ensure a complete reaction. Subsequently, the precipitate from different batches and solutions was separated using centrifugal equipment and dried to obtain the as-prepared black pigment precursors, which were calcined from room temperature to 1400 °C in an electric kiln (air, 1 h dwell time, 10 °C/min heating rate) in the next step. To investigate the color stability and composition at high temperatures, the obtained pigment powders were mixed with transparent glaze and pottery billet in ball milling equipment for 1 h, with a quality ratio of 2:1:1, and were calcined again at 1400 °C. The compositions of transparent glaze and pottery billet are presented in Table 2. Finally, the samples were coated on the billets, and the transparent glaze was sprayed on them. Following heat treatment at 1400 °C for a duration of 1 h, the coated tiles were subjected to colorimetric analysis. This method resulted in greater accuracy compared to naked-eye observations of pigments, irrespective of color alterations influenced by elements present in both glaze and billet constituents.

### 2.2. Characterization of Synthesized Pigments

The synthesized powders were characterized with thermogravimetry–differential scanning calorimetry (TG-DSC, STA 449 F5, Netzsch, Germany), X-ray photoelectron spectroscopy (XPS, Shimadzu, Japan), X-ray diffractometry (XRD, D/MAX2500VL/PC, Rigaku, Japan), and UV-Vis spectrophotometry (Shimadzu, Japan). The Rietveld refinement method was carried out using the Jade software package to analyze the crystal structure data. For the XRD results, the FWHM (full width with a half maximum) values were defined with reference to standard silicon. Debye–Scherrer’s equation was employed using peak widths to evaluate crystallite sizes, according to Equation (1) [22]. The cell parameters were calculated using the reflection planes (h k l) based on the formula for the cubic lattice (Equation (2)). The crystallinity degrees were determined using Equation (3) as follows:

(1) $$D=\frac{K\gamma }{B\cos\theta }$$

(2) $$a=\frac{\lambda }{2\cos\theta }\times \sqrt{h^{2}+k^{2}+l^{2}}$$

(3) $$X_{c}=\frac{I_{c}}{I_{c100}}\times 100\%$$

where *D* is the average thickness of the crystal grain perpendicular to the crystal plane, *K* is Scherrer constant, *γ* is the X-ray wavelength (generally 1.54056 Å), *B* is the half-height width or integral width of the measured sample diffraction peak, and *θ* is the Bragg angle in degrees. In Equation (2), *a* and *λ* represent the cell parameter and wavelength (Cu Ka), respectively [23]. In Equation (3), *I_c100_* is the integrated intensity of diffraction peaks and *I_c_* is the diffraction intensity of crystals.

The color performance of the obtained black pigments was measured using CIE-L*a*b* colorimetric parameters using a spectrophotometer (CM-700D, Konica, Japan). This colorimetric system measured for an illuminant D65, following the CIE-L*a*b* colorimetric method recommended by CIE (Commission Internationale del’Eclairage) [24]. L* represents lightness from 0 to 100, ranging from white to black and a* and b* are measures of chroma (−a* trends to green, +a* trends to red; −b* trends to blue, +b* trends to yellow).

Response surface methodology (RSM) was employed in the colorimetry analysis using Design-Expert version 7 (Design-Ease, Inc., Minneapolis, MN, USA). The optimization process was based on the Box–Behnken Design (BBD), and the statistical analysis consisted of 13 batches, which were also designed by the program according to the given formulations. The main aim was to investigate colorimetric transformation trends due to different ratios of Co, Fe, and Cr in black ceramic pigments. An analysis of variance (ANOVA) was used to assess the validity and accuracy of the RSM process.

## 3. Results and Discussion

### 3.1. Formulation Influences on Pigments

#### 3.1.1. Thermal Stability

**Figure 1:**
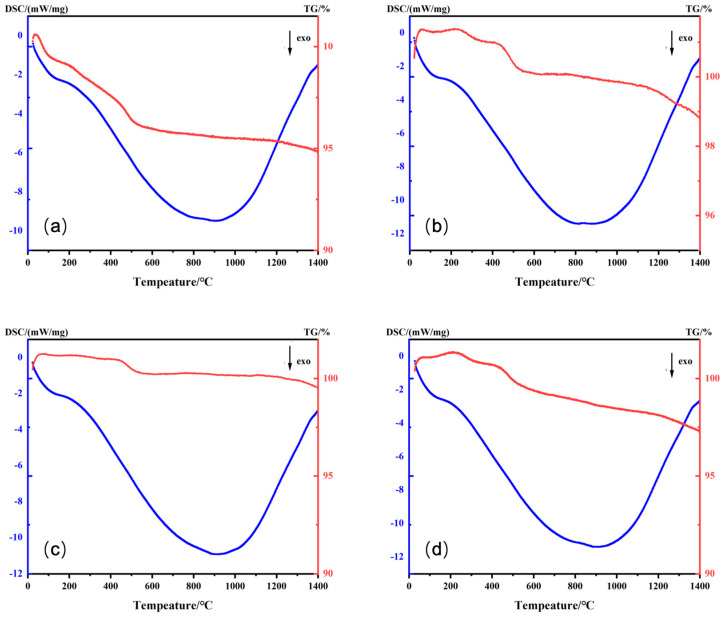
TG-DSC diagram of Sample (**a**) 3, (**b**) 6, (**c**) 11, and (**d**) 13.

In order to discuss the impact of the formulation on pigment structures, the chemical compositions of different samples were investigated. Before determining the terminal phases, a comprehensive thermal analysis was used to detect the stability of each sample. Among the samples, Sample 3, 6, 11, and 13 were typical and necessary to investigate further because they had either only one maximum or all maximum values in the proportions of Cr, Co, and/or Fe in the designed formulations. The TG-DSC thermal analysis of four samples sintered from room temperature to 1400 °C is shown in Figure 1. The TG curves of the four samples rose at a temperature below 100 °C, which indicates that CO_2_ in the air reacts with the precursors. At the same time, the DSC patterns indicate exothermic reactions occurring in all four samples. The weight loss observed in the temperature ranges of 0–100 °C, 100–400 °C, and 400–500 °C results from the loss of free water, constitution water from Cr(OH)_3_ and Fe(OH)_3_, and constitution water from Co(OH)_3_, respectively [25]. Among them, the weight loss observed for Sample 3 was the greatest, which aligns with its larger proportion of Co compared to the other samples. The quality of the four samples remained relatively stable and their exothermic reactions reached their peak at 900 °C. On the whole, the weight loss of the four samples corresponds with the formation of crystals, and the phases tend to remain unchanged over 900 °C [25].

The DSC curve of the 4 samples reached a minimum at 900 °C, which indicates that they probably have advantages for use in under-glazed ceramics at high temperatures. Additionally, the exothermic peaks are also related to particle size, with a larger peak area being consistent with tiny particles [26]. There was little difference in the exothermic peak areas of the four samples in Figure 1, so the elemental ratios have no obvious influence on the size of the particle. The four samples demonstrated weight loss phenomena as the temperature rose. The residual mass ratios were all above 94.83% and reached a peak of 99.51%, which reveals their outstanding high-temperature stability and suitability for use in ceramic products.

#### 3.1.2. Chemical Valences

**Figure 2:**
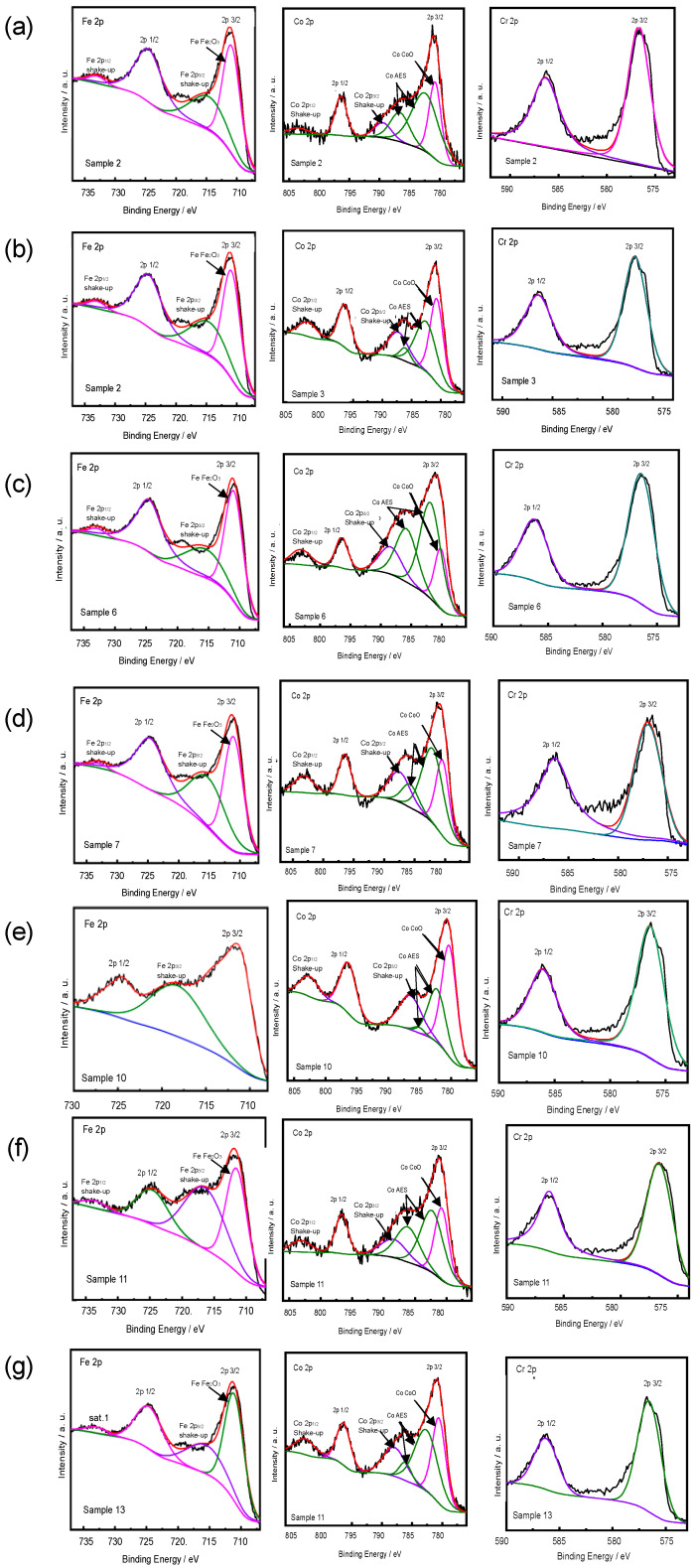
XPS spectra of Sample (**a**) 2, (**b**) 3, (**c**) 6, (**d**) 7, (**e**) 10, (**f**) 11, and (**g**) 13.

After the phases of the proposed samples were identified, it became significant to investigate their valence states because the elements involved (Cr, Fe, and Co) all have at least two valence states. Sample 2, 3, 6, 7, 10, 11, and 13 were characterized using XPS (Figure 2). The XPS results present the chemical environment of near-surface regions and relative atom ratios. In the spectra of chromium, the peaks at 576.73 and 588.37 eV correspond with Cr(III), which is also consistent with the spectra of chromium oxide (Cr_2_O_3_), as reported by Heiba et al. [27]. They also demonstrated bands of Fe(III) at 711.4 and 724.64 eV [28], similar to the results shown in Figure 2. Cobalt mainly appeared in the samples as Co(II), and the spectra were the same as those published in the literature [29,30]. The presence of a relatively narrow peak width and the inconspicuous satellite peaks on the high binding energy side of the 2p3/2 and 2p1/2 transitions suggest that only a limited fraction of Co^2+^ ions were situated within the octahedral positions within the crystallite lattice. To summarize, the XPS results show that Cr, Fe, and Co mainly exist with a single valence.

#### 3.1.3. Crystal Phases and Structures

**Figure 3:**
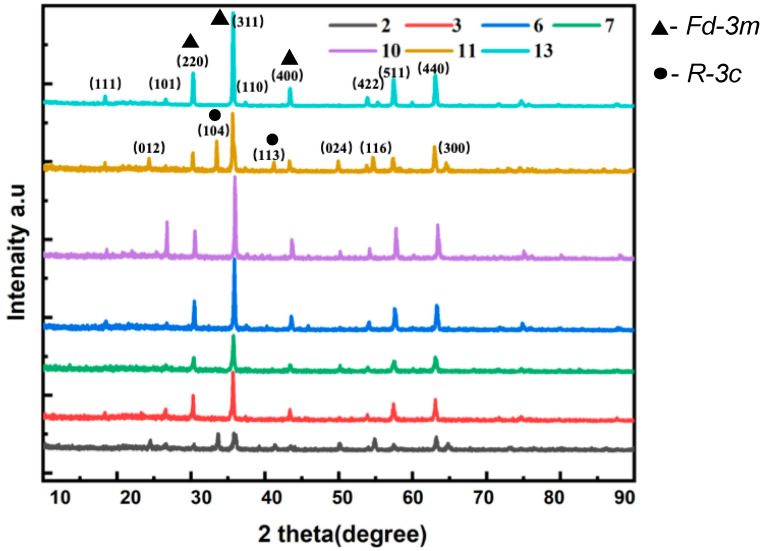
XRD diagrams of Sample 2, 3, 6, 7, 10, 11, and 13, with calcination at 1400 °C.

Based on the obtained valences of the different metal ions, the specific phase was researched further using XRD. The results of the seven selected samples with different ratios of Cr, Fe, and Co are illustrated in Figure 3. According to the JCPDS-ICDD cards [31], the main phase is the spinel structure (space group *Fd-3m*), which corresponds with the diffraction peaks of (311), (220), and (400) in each XRD pattern. The Co^2+^ ions exclusively occupy the tetrahedral sites in the spinel. Concurrently, Al^3+^ (from glaze and billet), Fe^3+^, and Cr^3+^ ions always take up the octahedral sites. The largest octahedral site stabilization energy (OSSE) of Cr^3+^ was around −195 kJ mol^−1^, making it preferentially occupy octahedral positions. Among these samples, the diffraction patterns of Sample 3, 7, 10, and 13 show a relatively pure phase of spinel. In addition, the presence of (101) crystalline surface in the spectrum of each sample corresponds with quartz, which is induced from glaze and billet during the preparation process.

**Figure 4:**
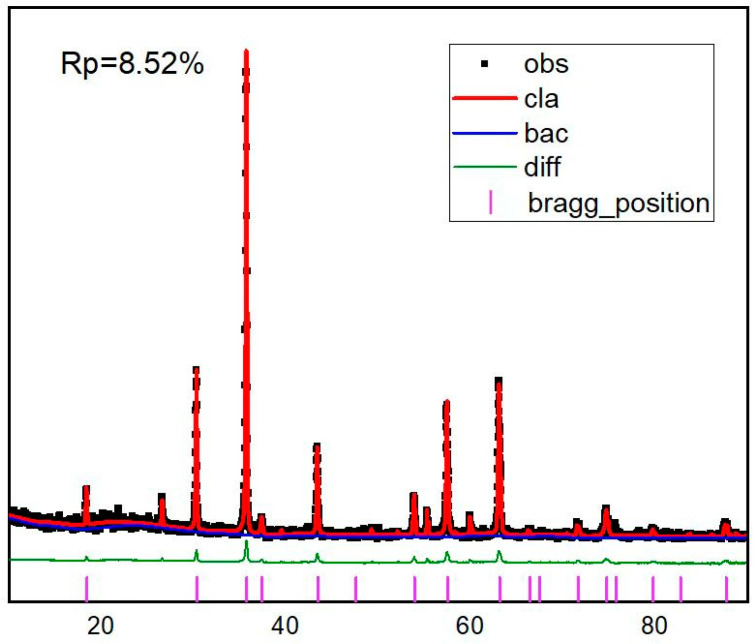
Rietveld analysis results for Sample 13, including fitting pattern, residual curve, Bragg reflection position, and R-factor.

However, there are still some interesting differences among the patterns due to different ratios. For Sample 2, a solid solution, (Cr_x_Fe_1–x_)_2_O_3_, is an obvious phase, as indicated by the peaks (104), (110), and (300), making it distinct from others but similar to Sample 11. The lattice parameters (a) of Sample 2 and 11 (space group *R-3c*) relative to (104), (110), and (300) were 4.9855 Å and 4.9992 Å, respectively, which fell between the values of Cr_2_O_3_ (chromium(III) oxide, eskolaite) (a = 4.957 Å) and Fe_2_O_3_ (iron(III) oxide, hematite) (a = 5.0206 Å), as determined from the Rietveld refinement results (Table 3). The fitting profiles and R-factor of Sample 13 are shown as an example in Figure 4. The identified phase of (Cr_x_Fe_1–x_)_2_O_3_ and its cell parameters were both investigated by Angeles et al. [32], and our results are consistent with their results when synthesizing spinel with a composition close to MgCr_2_O_4_.

Table 3 illustrates some mild variations in the crystalline data among the as-prepared samples, including lattice parameters, lattice volumes, crystallite sizes, crystallinity degrees, and the intensity ratio of diffraction peak (311) and (220). The lattice parameters a, b, and c of the crystal, with space group *Fd-3m*, were between 8.2865 and 8.3360 Å. Equivalently, the lattice volumes were all between 569.00 Å^3^ and 579.62 Å^3^. The crystal parameter range also indicates their composite spinel structures involving CoCr_2_O_4_ (a = 8.33 Å), CoFeCrO_4_ (a = 8.34 Å), and CoAl_2_O_4_ (a = 8.11 Å). In particular, Sample 10, which had the smallest lattice parameter, had a relatively larger amount of CoAl_2_O_4_ in its particles, which can make it appear blue. The spinel crystallite size ranged from 436 to 2743 Å, with Sample 10 exhibiting an exceptionally small size. It is worth mentioning that there is no relationship between the crystallite dimension and the grain size of the pigments [3]. Sample 10 and 13 demonstrated relatively better crystallinity and higher ratios of I(331) and I(220), which are associated with a better degree of reaction and higher cation mixing [33,34].

### 3.2. Chromatic Properties Analysis

**Figure 5:**
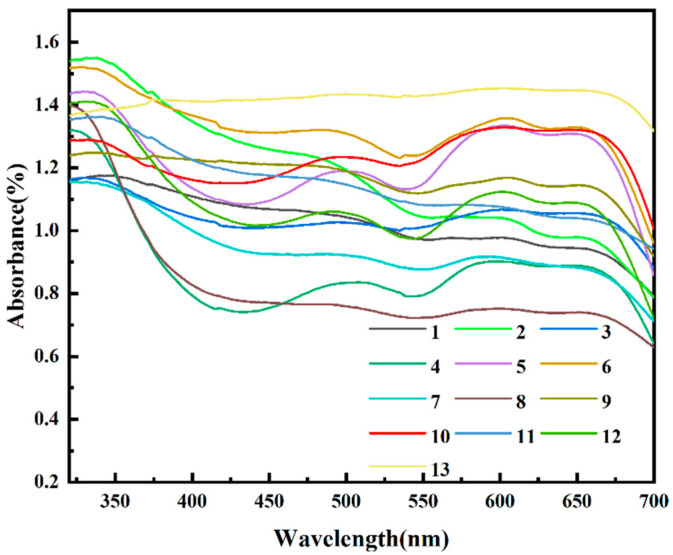
UV-Vis absorbance spectra of Samples 1–13 in under-glazed porcelain tiles sintered at 1400 °C.

The chromatic properties of black pigments synthesized with different formulations vary from each other, owing to the differences in phases and crystal structures. UV-Vis spectroscopy (Figure 5) describes the absorption bands of Samples 1–13 in under-glazed porcelain tiles sintered at 1400 °C. All the absorbance spectra revealed similar features generally, but there were still important variations across several wavelength regions, and the absorbance values were distinguished from each other due to the varying ratios of metallic oxide used in the preparations. The color coordinate values L*, a*, and b* of the under-glazed tiles coated with Samples 1–13 are shown in Table 4, providing an alternative perspective on their chromatic properties.

The most conspicuous curve of Sample 13 presents high absorbance across the entire wavelength region. This indicates that the color of this sample exhibited a high-intensity black hue, in line with the extremely low value of L* (4.26) and both a* and b* values, which approached zero, as shown in Table 4. Rives et al. [35] also focused on black ceramic pigments, and they reported that high-quality black pigments have low L*, a*, and b* values that approach zero. The spectra of Samples 3, 4, 5, 6, 10, and 12 had low absorbance in the wavelength region of 400–530 nm and strong absorbance in the 580–670 nm range, implying that these samples reflect the counter color of green–blue. Similarly, the a* values of these pigments were all negative, indicating a green color, and the b* values corresponded with blue. The results indicate that there were more Co^2+^ ions in tetrahedral positions and more Cr^3+^ ions in octahedral positions, which contribute to the formation of stable spinel structures. In addition, the refined lattice parameters suggest that spinel CoAl_2_O_4_ was the main phase in Sample 10, which is consistent with the most negative b* value in the analysis. The same results were also obtained by Lei et al. previously [36]. It can be seen in Table 4 that Samples 1, 2, and 11 have obvious large values for both a* and b*, representing the colors red and yellow, respectively. The relatively low absorbances in the yellow (580–600 nm) and red (600–700 nm) ranges indicate that the color tends towards yellow–red. In addition, each spectrum shows an absorption peak at 600 nm, which is related to the presence of Co^2+^ ions occupying tetrahedral sites [37], which was also confirmed by the XRD results mentioned above.

### 3.3. Formulation Optimization

As can be seen in Table 1 and Table 4, there is not a specific linear relationship between the color coordinate parameters and the proportions of Cr, Co, and Fe. Thus, to further investigate the colorimetric trends based on different formulations, the color coordinate values L*, a*, and b* are discussed using statistical models through RSM. The three-dimensional (3D) models were established according to the color coordinate values, the contents of Cr, Co, and Fe, and their functional relevance, which were based on mathematical equations derived from fundamental parameters. Table 5, Table 6 and Table 7 show the ANOVA used to evaluate the cubic modeling of the colorimetric parameters L*, a*, and b*, respectively. A, B, and C in these three tables represent the atomic ratios of Cr, Co, and Fe in each batch. For the models to fit, significant model terms (e.g., A, A^2^, and A^2^B) were selected with their relative coefficients. The F-values were all large enough and the *p*-values were lower than 0.05, which means that the models are statistically significant. The coefficient of determination (R^2^) and the adjusted coefficient of determination (adjusted R^2^) were close, suggesting that the models can effectively guide the design process.

According to the influence of each factor on the sensitivity and significance of CIE- L*a*b*, the coded equations (Equations (4)–(6)) related to the L*, a*, and b* values were obtained using regression analysis [38,39]:

L* = 4.26 − 1.28 *B* + 4.13 *C* + 1.33 *AB* + 2.27 *BC* + 3.71 *A*^2^ + 4.54 *B*^2^ + 5.22 *C*^2^ − 3.55 *A*^2^*B* (4)

a* = −0.0514 − 0.1325 *A* − 1.58 *B* + 0.11 *C* − 0.4975 *AC* − 1.37 *BC* + *B*^2^ − 1.84 *C*^2^ + 1.52 *A*^2^*C* + 0.4075 *AB*^2^ (5)

b* = −0.1040 + 2.75 A − 5.64 B + 2.31 C + 2.13 A^2^ − 4.61 AB^2^ (6)

**Figure 6:**
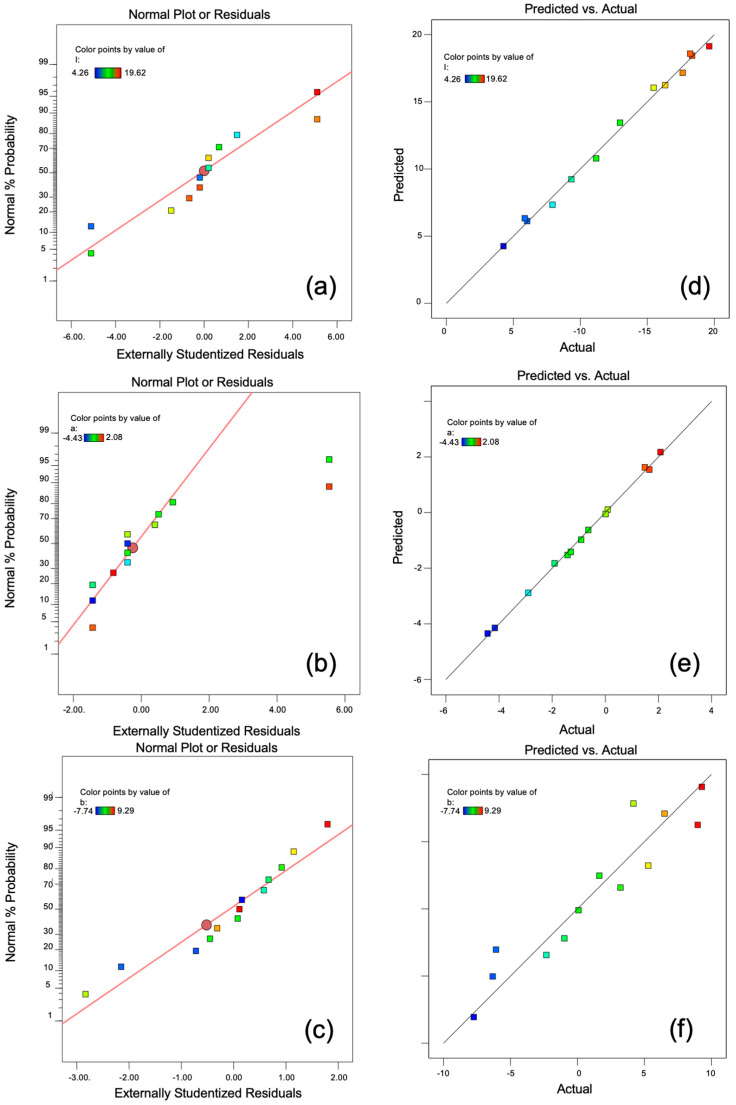
Residual distribution of values (**a**) L*, (**b**) a*, and (**c**) b*; predicted versus actual results for color coordinate values (**d**) L*, (**e**) a*, and (**f**) b*.

Figure 6a–c shows the residual distribution of each color coordinate value, and Figure 6d–f compares the predicted data with the actual data of L*, a*, and b*. As can be seen, the values are close to the straight lines because the experimental results are in accordance with the designed model. Thus, the model system is useful to design, predict, and optimize the synthesis of black ceramic pigments.

**Figure 7:**
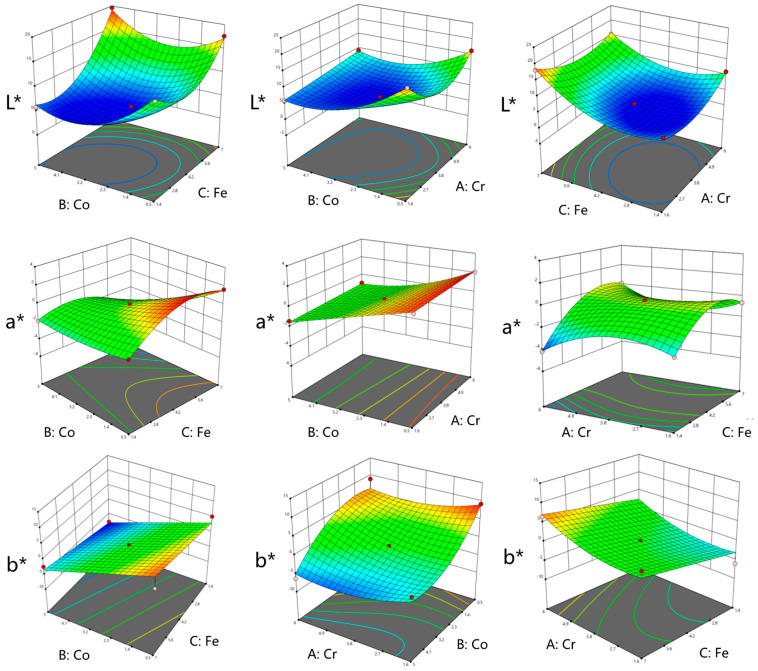
The 3D response surface plots for color coordinate values L*, a*, and b*.

The 3D response surface and counter maps for the colorimetry values versus the affecting factors of different formulations are shown in Figure 7. The variables of the response surfaces were constructed based on Sample 13 (n(Cr):n(Co):n(Fe) = 3.8:2.75:4.2) and the response surfaces revealed the colorimetric trends when the proportion of tinted metal ions changed. According to the nine surfaces, the L*, a*, and b* values were set as constraints and were set to approach 0, which is indicative of high-quality black ceramic pigments. Four suggested groups of predicted optimized black pigment formulations and their corresponding color coordinate values are illustrated in Table 8. Each one of them has better colorimetry properties than Sample 13, which had the lowest L* value of all the groups. The intensity of the black color is mainly influenced by the L* value, although there are a few variations in the absolute values of a*.

## 4. Conclusions

In summary, thirteen samples with different proportions were prepared using the co-precipitation method and characterized with XRD, XPS, UV, TG-DSC, and colorimetry analyses. The synthesized Cr-Co-Fe system black pigments were mainly composed of spinel crystals, including CoCr_2_O_4_, CoFeCrO_4_, and CoAl_2_O_4_. Some of the as-synthesized pigments also showed an additional *R-3c* space group when the contents of Cr and Fe were excessive. Furthermore, the colorimetry analysis included color coordinate values L*, a*, and b*, and RSM was applied. The results of the ANOVA evaluations confirmed the validity and accuracy of the 3D models. Based on the statistical relationships between the proportions of Cr, Co, and Fe and the color coordinate values, several optimized solutions were suggested for purer and more intense black pigments. The color coordinate values of the optimized pigment are L* = 3.741, a* = −0.125, and b* = 0, with a ratio of Cr, Co, and Fe equal to 4.003:2.591:3.515, which possesses a wide range of adjustable colors. The statistical relationships established in this study can be useful for designing, predicting, and controlling the relatively precious color properties of Cr-Co-Fe system black pigments, and they may also be relevant for other corresponding pigments.

## Figures and Tables

**Table 1:** The mole ratio of Cr, Co, and Fe in 13 batches.

Sample	Cr	Co	Fe
1	1.6	0.5	4.2
2	6	0.5	4.2
3	1.6	5	4.2
4	6	5	4.2
5	1.6	2.75	1.4
6	6	2.75	1.4
7	1.6	2.75	7
8	6	2.75	7
9	3.8	0.5	1.4
10	3.8	5	1.4
11	3.8	0.5	7
12	3.8	5	7
13	3.8	2.75	4.2

**Table 2:** Compositions (wt%) of transparent glaze and pottery billet.

wt%	SiO_2_	Al_2_O_3_	CaO	Fe_2_O_3_	Na_2_O	TiO_2_	MgO	K_2_O
Glaze	67.20	13.10	6.64	0.22	5.85	0.02	0.99	5.24
Billet	71.34	14.89	0.12	7.45	0.33	0.88	1.00	2.99

**Table 3:** Crystal structure parameters of the as-prepared particles, including the lattice parameter, lattice volume, crystallite size, crystallinity, and I_(331)_/I_(220)_.

Sample	*Fd-3m*		*R-3c*			Crystallite Size (Å)	Crystallinity (%)	I_(311)_ /I_(220)_
	Lattice Parameter a, b, and c (Å)	Lattice Volume (Å^3^)	Lattice Parameter a and b (Å)	Lattice Parameter c (Å)	Lattice Volume (Å^3^)			
2	8.3205	576.00	4.9855	13.5115	290.83	782	86.44	1.11
3	8.3311	578.25				1745	85.39	1.97
6	8.3211	576.16				705	82.07	2.50
7	8.3293	577.64				781	84.81	2.67
10	8.2865	569.00				436	96.42	2.82
11	8.3360	579.26	4.9992	13.5914	293.92	2743	86.02	2.06
13	8.3310	578.21				1056	96.56	2.87

**Table 4:** The color coordinate values of Samples 1–13 in under-glazed porcelain tiles sintered at 1400 °C.

Sample	L*	a*	b*
1	18.34	1.49	9.29
2	16.33	2.08	8.98
3	6.03	−1.42	−0.98
4	9.33	−0.91	−6.33
5	7.92	−2.9	−6.08
6	11.18	−4.16	−1.63
7	18.20	−0.64	3.21
8	15.47	0.09	6.5
9	12.96	−1.3	5.28
10	5.85	−1.91	−7.74
11	17.64	1.66	4.18
12	19.62	−4.43	−2.32
13	4.26	0.01	0.08

**Table 5:** ANOVA evaluation data for the cubic modeling of color coordinate value L*.

Source	Sum of Squares	Df	Mean Square	F-Value	*p*-Value	
Model	342.79	8	42.85	15.12	0.0096	significant
B-Co	6.58	1	6.58	2.32	0.2023	
C-Fe	136.29	1	136.29	48.08	0.0023	
AB	7.05	1	7.05	2.49	0.1899	
BC	20.66	1	20.66	7.29	0.0541	
A^2^	31.48	1	31.48	11.11	0.0290	
B^2^	47.03	1	47.03	16.59	0.0152	
C^2^	62.31	1	62.31	21.98	0.0094	
A^2^B	25.13	1	25.13	8.87	0.0408	
Residual	11.34	4	2.83			
Cor Total	354.13	12				
Standard deviation	1.68	R^2^		0.9680		
Mean	12.55	Adjusted R^2^		0.9039		
Coefficient variation %	13.42	Predicted R^2^		0.8368		
	Adequacy Precision			10.6186		

**Table 6:** ANOVA evaluation data for the cubic modelling of color coordinate value a*.

Source	Sum of Squares	df	Mean Square	F-Value	*p*-Value	
Model	51.44	9	5.72	194.40	0.0005	significant
A-Cr	0.0702	1	0.0702	2.39	0.2199	
B-Co	19.84	1	19.84	674.97	0.0001	
C-Fe	0.0484	1	0.0484	1.65	0.2896	
AC	0.9900	1	0.9900	33.67	0.0102	
BC	7.51	1	7.51	255.35	0.0005	
B^2^	0.3975	1	0.3975	13.52	0.0348	
C^2^	9.44	1	9.44	320.92	0.0004	
A^2^C	4.61	1	4.61	156.65	0.0011	
AB^2^	0.3321	1	0.3321	11.30	0.0437	
Residual	0.0882	3	0.0294			
Cor Total	51.53	12				
Standard deviation	0.1715	R^2^		0.9983		
Mean	−0.9492	Adjusted R^2^		0.9932		
Coefficient variation %	18.06	Predicted R^2^		0.9498		
	Adequacy Precision			43.3595		

**Table 7:** ANOVA evaluation data for the cubic modelling of color coordinate value b*.

Source	Sum of Squares	df	Mean Square	F-Value	*p*-Value	
Model	349.18	5	69.84	11.46	0.0029	significant
A-Cr	30.25	1	30.25	4.96	0.0612	
B-Co	254.25	1	254.25	41.72	0.0003	
C-Fe	42.69	1	42.69	7.00	0.0331	
A^2^	13.98	1	13.98	2.29	0.1737	
AB^2^	34.69	1	34.69	5.69	0.0485	
Residual	42.66	7	6.09			
Cor Total	391.84	12				
Standard deviation	2.47	R^2^		0.8911		
Mean	1.21	Adjusted R^2^		0.8134		
Coefficient variation %	204.41	Predicted R^2^		0.5984		
	Adequacy Precision			10.2149		

**Table 8:** The suggested parameters of optimized black ceramic pigments.

Number	Cr	Co	Fe	L*	a*	b*	Desirability
1	4.003	2.591	3.515	3.741	−0.125	0.000	0.769
2	4.020	2.597	3.504	3.734	−0.136	0.000	0.769
3	4.049	2.619	3.520	3.729	−0.146	0.000	0.768
4	4.081	2.638	3.521	3.723	−0.161	0.000	0.768

## Data Availability

All relevant data are within the paper.
